# [(4*E*)-1-Methyl-2,6-diphenyl-3-(propan-2-yl)piperidin-4-yl­idene]amino 3-methyl­benzoate

**DOI:** 10.1107/S160053681301893X

**Published:** 2013-07-20

**Authors:** T. Vinuchakkaravarthy, R. Sivakumar, T. Srinivasan, V. Thanikachalam, D. Velmurugan

**Affiliations:** a Centre of Advanced Study in Crystallography and Biophysics, University of Madras, Maraimalai (Guindy) Campus, Chennai 600 025, India; b Department of Chemistry, Annamalai University, Annamalai Nagar, Chidambaram 608 002, India

## Abstract

In the title compound, C_29_H_32_N_2_O_2_, the piperidine ring exists in a chair conformation (the bond-angle sum at the *sp* ^2^-hybridized C atom is 359.79°). The phenyl rings and the methyl group substituted on the heterocyclic ring are in equatorial orientations. In the crystal, pairs of C—H⋯π inter­actions result in the formation of inversion dimers.

## Related literature

For the synthesis and the biological activity of piperidinyl-4-one derivatives, see: Parthiban *et al.* (2009 ▶, 2011 ▶). For the crystal structures of related compounds, see: Park *et al.* (2012*a* ▶,*b* ▶). For ring puckering parameters, see: Cremer & Pople (1975 ▶).
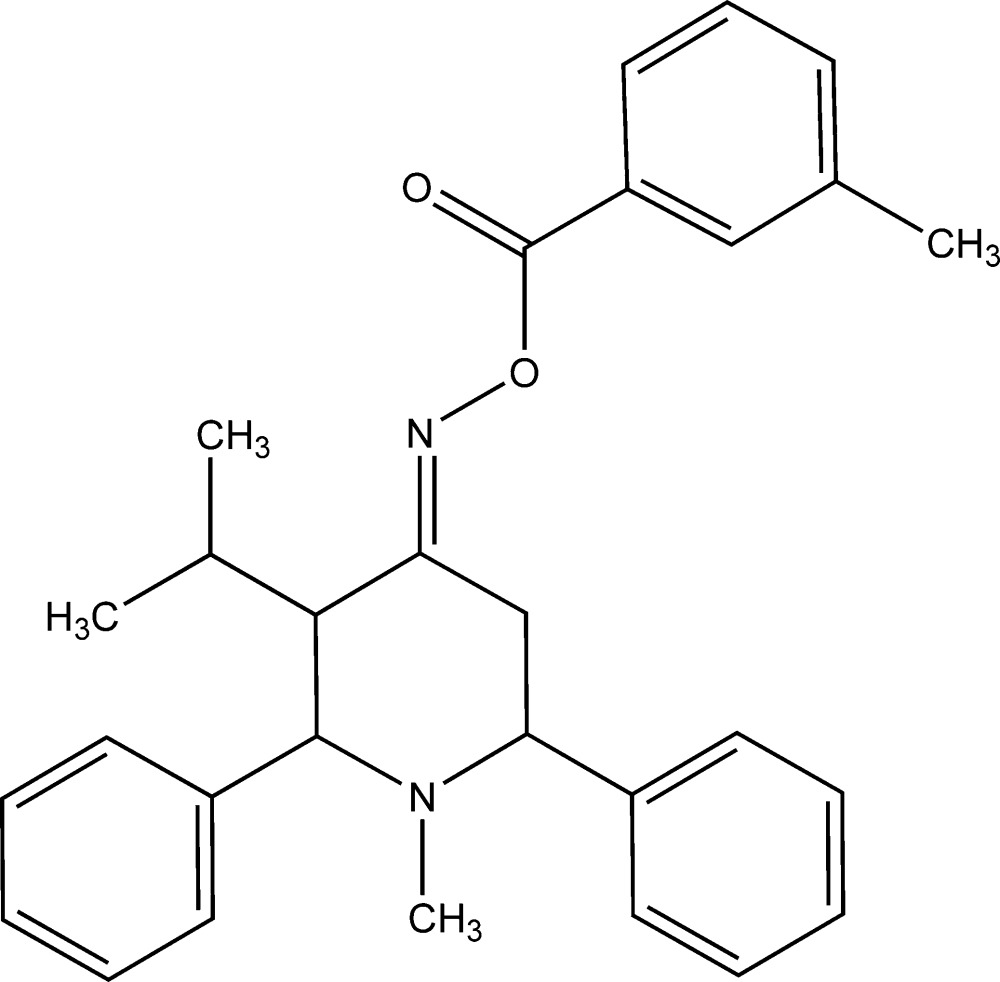


## Experimental

### 

#### Crystal data

C_29_H_32_N_2_O_2_
*M* *_r_* = 440.57
Triclinic, 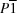

*a* = 10.7837 (4) Å
*b* = 11.7075 (4) Å
*c* = 12.0586 (4) Å
α = 114.352 (3)°
β = 96.245 (2)°
γ = 109.530 (5)°
*V* = 1252.07 (10) Å^3^
*Z* = 2
Mo *K*α radiation
μ = 0.07 mm^−1^
*T* = 293 K
0.20 × 0.20 × 0.20 mm

#### Data collection

Bruker SMART APEXII CCD diffractometer
Absorption correction: multi-scan (*SADABS*; Bruker, 2008 ▶) *T*_min_ = 0.986, *T*_max_ = 0.986
18725 measured reflections
5163 independent reflections
3847 reflections with *I* > 2σ(*I*)
*R*_int_ = 0.028

#### Refinement

*R*[*F* ^2^ > 2σ(*F* ^2^)] = 0.047
*wR*(*F* ^2^) = 0.144
*S* = 1.04
5163 reflections
298 parameters
H-atom parameters constrained
Δρ_max_ = 0.19 e Å^−3^
Δρ_min_ = −0.20 e Å^−3^

### 

Data collection: *APEX2* (Bruker, 2008 ▶); cell refinement: *SAINT* (Bruker, 2008 ▶); data reduction: *SAINT*; program(s) used to solve structure: *SHELXS97* (Sheldrick, 2008 ▶); program(s) used to refine structure: *SHELXL97* (Sheldrick, 2008 ▶); molecular graphics: *ORTEP-3 for Windows* (Farrugia, 2012 ▶); software used to prepare material for publication: *SHELXL97* and *PLATON* (Spek, 2009 ▶).

## Supplementary Material

Crystal structure: contains datablock(s) global, I. DOI: 10.1107/S160053681301893X/pv2632sup1.cif

Structure factors: contains datablock(s) I. DOI: 10.1107/S160053681301893X/pv2632Isup2.hkl

Supplementary material file. DOI: 10.1107/S160053681301893X/pv2632Isup3.cml

Additional supplementary materials:  crystallographic information; 3D view; checkCIF report

## Figures and Tables

**Table 1:** Hydrogen-bond geometry (Å, °) *Cg*1 is the centroid of the C16–C21 ring.

*D*—H⋯*A*	*D*—H	H⋯*A*	*D*⋯*A*	*D*—H⋯*A*
C11—H11⋯*Cg*1^i^	0.93	2.95	3.778 (2)	149

Symmetry code: (i) 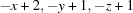.

**Fig. 1.**
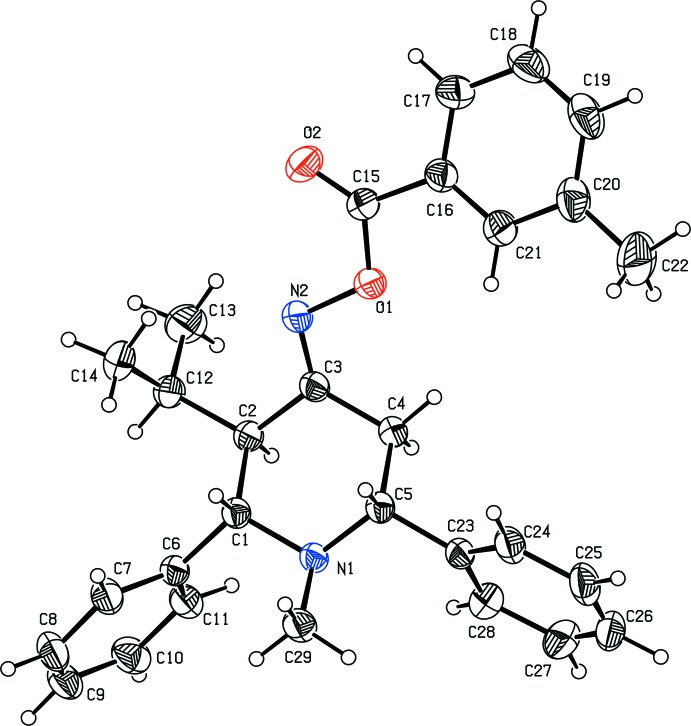
The molecular structure of the title compound with the atom numbering scheme. Displacement ellipsoids are drawn at the 30% probability level. H atoms are presented as small spheres of arbitrary radius.

**Fig. 2.**
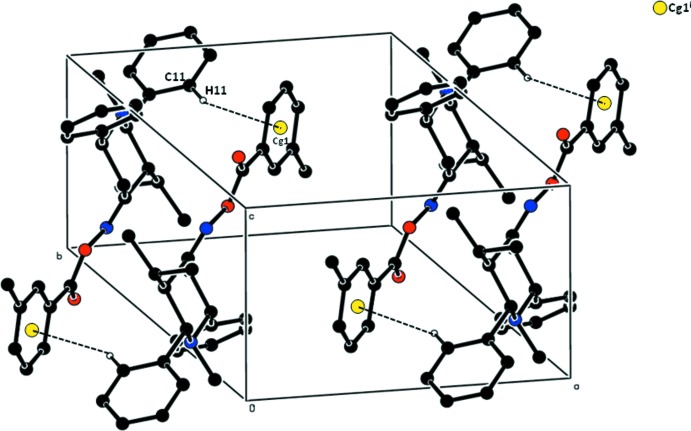
The crystal packing arrangement of the title compound viewed down the b axis showing intermolecular C—H···Cg hydrogen bond interaction (dashed line).

## supplementary crystallographic information

### Comment

Piperdin-4-one nucleus is an important pharmacophore due to its broad spectrum of biological actions ranging from antibacterial to anticancer (Parthiban *et al.*, 2009; 2011). Hence, the synthesis and steriochemical analysis of piperdin-4-one nucleus based pharmacophores has gained much interest in the field of medicinal chemistry.

The bond distances and angles in the title compound (Fig. 1) agree very well with the corresponding bond distances and angles reported in closely related compounds (Park *et al.*, 2012*a*; 2012*b*). The piperidone ring (N1/C1—/C5) adopts a chair conformation with puckering parameters: *Q* =0.575 (2) Å, θ = 174.3 (2)° and φ = 359 (2)° (Cremer & Pople, 1975). The molecule exists in its E-isomeric form.

The packing of the molecules within the crystal is shown in Fig. 2. The crystal structure is stabilized by intermolecular C11—H11···*Cg*1^i^ hydrogen bonding interactions resulting in centrosymmetric dimers about inversion centers, where *Cg*1 is the center of gravity of the ring atoms (C16—C21) (Table 1).

### Experimental

3-Isopropyl-2,6-diphenylpiperidin-4-one was synthesized by Mannich condensation using benzaldehyde (2 mol), ammonium acetate (1 mol) and isobutyl methyl ketone (1 mol) in absolute ethanol, warmed for 30 min and stirred overnight at room temperature. The product was treated with methyl iodide (1.5 mol) in the presence of potassium carbonate (2 mol) in acetone (10 ml) and refluxed for two hours yielding (4E)-1-methyl-3-isopropyl-2,6-diphenylpiperidin -4-one. The oximation was done by hydroxylamine hydrochloride (2 mol) in the presence of sodium acetate (2 mol) in ethanol (10 ml) and refluxed for two hours. The resulting oxime (0.5 g, 1.55 mmol) was stirred with dry pyridine (5 ml), added 3-methylbenzoic acid (0.23 g, 1.7 mmol) followed by the dropwise addition of phosphorus oxychloride (0.21 mL, 2.3 mmol) and stirred at ambient temperature for 15 min; the progress of the reaction was monitored by thin layer chromatography. Upon completion of the reaction, saturated sodium bicarbonate solution (8 ml) was added to the reaction mixture. The product was filtered and dried to get a white solid (0.62 g, 91%) which was recrystallized from ethanol to yield crystals suitable for X-ray crystallographic studies.

### Refinement

H atoms were positioned geometrically (C—H = 0.93–0.98 Å) and allowed to ride on their parent atoms, with *U*_iso_(H) = 1.5*U*_eq_(C) for methyl H and 1.2*U*_eq_(C) for other H atoms.

### Crystal data

**Table:**

C_29_H_32_N_2_O_2_	*Z* = 2
*M**_r_* = 440.57	*F*(000) = 472
Triclinic, *P*1	*D*_x_ = 1.169 Mg m^−^^3^
Hall symbol: -P 1	Mo *K*α radiation, λ = 0.71073 Å
*a* = 10.7837 (4) Å	Cell parameters from 5207 reflections
*b* = 11.7075 (4) Å	θ = 1.9–26.5°
*c* = 12.0586 (4) Å	µ = 0.07 mm^−^^1^
α = 114.352 (3)°	*T* = 293 K
β = 96.245 (2)°	Block, colorless
γ = 109.530 (5)°	0.20 × 0.20 × 0.20 mm
*V* = 1252.07 (10) Å^3^	

### Data collection

**Table:**

Bruker SMART APEXII CCD diffractometer	5163 independent reflections
Radiation source: fine-focus sealed tube	3847 reflections with *I* > 2σ(*I*)
Graphite monochromator	*R*_int_ = 0.028
ω and φ scans	θ_max_ = 26.6°, θ_min_ = 1.9°
Absorption correction: multi-scan (*SADABS*; Bruker, 2008)	*h* = −13→13
*T*_min_ = 0.986, *T*_max_ = 0.986	*k* = −14→14
18725 measured reflections	*l* = −15→15

### Refinement

**Table:**

Refinement on *F*^2^	Primary atom site location: structure-invariant direct methods
Least-squares matrix: full	Secondary atom site location: difference Fourier map
*R*[*F*^2^ > 2σ(*F*^2^)] = 0.047	Hydrogen site location: inferred from neighbouring sites
*wR*(*F*^2^) = 0.144	H-atom parameters constrained
*S* = 1.04	*w* = 1/[σ^2^(*F*_o_^2^) + (0.0698*P*)^2^ + 0.2364*P*] where *P* = (*F*_o_^2^ + 2*F*_c_^2^)/3
5163 reflections	(Δ/σ)_max_ < 0.001
298 parameters	Δρ_max_ = 0.19 e Å^−^^3^
0 restraints	Δρ_min_ = −0.20 e Å^−^^3^

### Special details

**Table:**

Geometry. All e.s.d.'s (except the e.s.d. in the dihedral angle between two l.s. planes) are estimated using the full covariance matrix. The cell e.s.d.'s are taken into account individually in the estimation of e.s.d.'s in distances, angles and torsion angles; correlations between e.s.d.'s in cell parameters are only used when they are defined by crystal symmetry. An approximate (isotropic) treatment of cell e.s.d.'s is used for estimating e.s.d.'s involving l.s. planes.
Refinement. Refinement of *F*^2^ against ALL reflections. The weighted *R*-factor *wR* and goodness of fit *S* are based on *F*^2^, conventional *R*-factors *R* are based on *F*, with *F* set to zero for negative *F*^2^. The threshold expression of *F*^2^ > σ(*F*^2^) is used only for calculating *R*-factors(gt) *etc*. and is not relevant to the choice of reflections for refinement. *R*-factors based on *F*^2^ are statistically about twice as large as those based on *F*, and *R*- factors based on ALL data will be even larger.

### Fractional atomic coordinates and isotropic or equivalent isotropic displacement parameters (Å^2^)

**Table:**

	*x*	*y*	*z*	*U*_iso_*/*U*_eq_	
C1	0.85940 (14)	0.07747 (14)	0.32666 (13)	0.0429 (3)	
H1	0.9292	0.0507	0.3537	0.051*	
C2	0.86023 (15)	0.20135 (15)	0.44415 (13)	0.0453 (3)	
H2	0.7928	0.2279	0.4125	0.054*	
C3	0.99781 (15)	0.31978 (15)	0.48597 (14)	0.0467 (3)	
C4	1.02338 (17)	0.35945 (15)	0.38514 (14)	0.0506 (4)	
H4A	1.1116	0.4378	0.4169	0.061*	
H4B	0.9527	0.3856	0.3610	0.061*	
C5	1.02226 (15)	0.23655 (15)	0.26978 (14)	0.0441 (3)	
H5	1.0983	0.2159	0.2947	0.053*	
C6	0.72125 (15)	−0.04589 (15)	0.27061 (14)	0.0457 (3)	
C7	0.71016 (18)	−0.17315 (17)	0.25442 (17)	0.0593 (4)	
H7	0.7883	−0.1828	0.2817	0.071*	
C8	0.5842 (2)	−0.28672 (18)	0.19804 (19)	0.0709 (5)	
H8	0.5786	−0.3721	0.1865	0.085*	
C9	0.4681 (2)	−0.27374 (19)	0.15942 (19)	0.0715 (5)	
H9	0.3836	−0.3499	0.1219	0.086*	
C10	0.47680 (18)	−0.1480 (2)	0.17625 (18)	0.0676 (5)	
H10	0.3977	−0.1386	0.1513	0.081*	
C11	0.60263 (16)	−0.03497 (17)	0.23022 (16)	0.0555 (4)	
H11	0.6076	0.0495	0.2395	0.067*	
C12	0.81213 (17)	0.16674 (17)	0.54654 (15)	0.0562 (4)	
H12	0.7193	0.0924	0.5043	0.067*	
C13	0.7980 (2)	0.2888 (2)	0.64964 (19)	0.0817 (6)	
H13A	0.7437	0.3201	0.6108	0.123*	
H13B	0.8874	0.3626	0.6981	0.123*	
H13C	0.7540	0.2606	0.7048	0.123*	
C14	0.8961 (2)	0.1127 (2)	0.60377 (19)	0.0763 (6)	
H14A	0.9018	0.0352	0.5370	0.114*	
H14B	0.8528	0.0845	0.6592	0.114*	
H14C	0.9868	0.1843	0.6513	0.114*	
C15	1.29265 (17)	0.54898 (17)	0.72791 (15)	0.0541 (4)	
C16	1.41632 (16)	0.65991 (17)	0.73437 (15)	0.0533 (4)	
C17	1.49678 (19)	0.7698 (2)	0.85301 (18)	0.0671 (5)	
H17	1.4752	0.7713	0.9261	0.081*	
C18	1.6094 (2)	0.8772 (2)	0.8616 (2)	0.0790 (6)	
H18	1.6626	0.9521	0.9408	0.095*	
C19	1.64325 (19)	0.8744 (2)	0.7553 (2)	0.0780 (6)	
H19	1.7197	0.9475	0.7632	0.094*	
C20	1.56626 (19)	0.7648 (2)	0.6353 (2)	0.0734 (5)	
C21	1.45135 (18)	0.65867 (19)	0.62697 (18)	0.0629 (4)	
H21	1.3967	0.5852	0.5474	0.075*	
C22	1.6026 (3)	0.7604 (3)	0.5167 (3)	0.1156 (9)	
H22A	1.6847	0.8408	0.5396	0.173*	
H22B	1.5287	0.7586	0.4629	0.173*	
H22C	1.6176	0.6790	0.4722	0.173*	
C23	1.04536 (16)	0.27730 (15)	0.16717 (14)	0.0480 (4)	
C24	1.17432 (18)	0.31908 (16)	0.15031 (17)	0.0592 (4)	
H24	1.2461	0.3181	0.1996	0.071*	
C25	1.1978 (3)	0.36252 (19)	0.0605 (2)	0.0777 (6)	
H25	1.2851	0.3895	0.0496	0.093*	
C26	1.0947 (3)	0.3661 (2)	−0.0117 (2)	0.0870 (7)	
H26	1.1111	0.3957	−0.0715	0.104*	
C27	0.9668 (3)	0.3257 (2)	0.0046 (2)	0.0851 (6)	
H27	0.8959	0.3280	−0.0446	0.102*	
C28	0.9410 (2)	0.28125 (19)	0.09366 (17)	0.0653 (5)	
H28	0.8533	0.2541	0.1037	0.078*	
C29	0.90196 (18)	−0.00294 (16)	0.12275 (16)	0.0566 (4)	
H29A	0.8183	−0.0841	0.0931	0.085*	
H29B	0.9777	−0.0182	0.1534	0.085*	
H29C	0.9159	0.0168	0.0541	0.085*	
N1	0.89281 (12)	0.11408 (12)	0.22587 (11)	0.0422 (3)	
N2	1.08029 (13)	0.36687 (14)	0.59413 (12)	0.0536 (3)	
O1	1.20675 (11)	0.47558 (12)	0.60903 (10)	0.0601 (3)	
O2	1.26989 (15)	0.52897 (16)	0.81397 (12)	0.0881 (5)	

### Atomic displacement parameters (Å^2^)

**Table:**

	*U*^11^	*U*^22^	*U*^33^	*U*^12^	*U*^13^	*U*^23^
C1	0.0428 (8)	0.0407 (8)	0.0444 (8)	0.0152 (6)	0.0146 (6)	0.0211 (6)
C2	0.0459 (8)	0.0444 (8)	0.0426 (8)	0.0152 (6)	0.0162 (6)	0.0203 (7)
C3	0.0517 (9)	0.0385 (8)	0.0429 (8)	0.0140 (7)	0.0173 (7)	0.0163 (6)
C4	0.0594 (9)	0.0396 (8)	0.0441 (8)	0.0118 (7)	0.0155 (7)	0.0194 (7)
C5	0.0428 (8)	0.0427 (8)	0.0460 (8)	0.0145 (6)	0.0150 (6)	0.0227 (7)
C6	0.0480 (8)	0.0413 (8)	0.0422 (8)	0.0128 (6)	0.0169 (6)	0.0189 (6)
C7	0.0619 (10)	0.0486 (9)	0.0650 (10)	0.0182 (8)	0.0192 (8)	0.0288 (8)
C8	0.0805 (13)	0.0423 (9)	0.0783 (12)	0.0124 (9)	0.0245 (10)	0.0287 (9)
C9	0.0611 (11)	0.0523 (10)	0.0703 (12)	−0.0010 (8)	0.0195 (9)	0.0215 (9)
C10	0.0495 (10)	0.0627 (11)	0.0708 (11)	0.0123 (8)	0.0141 (8)	0.0241 (9)
C11	0.0523 (9)	0.0457 (9)	0.0584 (9)	0.0144 (7)	0.0149 (7)	0.0212 (8)
C12	0.0529 (9)	0.0560 (9)	0.0473 (8)	0.0091 (7)	0.0193 (7)	0.0235 (8)
C13	0.0864 (14)	0.0854 (14)	0.0633 (12)	0.0285 (12)	0.0426 (11)	0.0278 (11)
C14	0.0871 (14)	0.0762 (13)	0.0683 (12)	0.0196 (11)	0.0222 (10)	0.0485 (11)
C15	0.0545 (9)	0.0547 (9)	0.0462 (8)	0.0158 (8)	0.0118 (7)	0.0243 (8)
C16	0.0471 (9)	0.0518 (9)	0.0548 (9)	0.0148 (7)	0.0074 (7)	0.0264 (8)
C17	0.0614 (11)	0.0642 (11)	0.0579 (10)	0.0168 (9)	0.0018 (8)	0.0247 (9)
C18	0.0592 (12)	0.0597 (12)	0.0859 (14)	0.0090 (9)	−0.0089 (10)	0.0263 (11)
C19	0.0489 (10)	0.0653 (12)	0.1112 (18)	0.0103 (9)	0.0090 (11)	0.0490 (13)
C20	0.0564 (11)	0.0755 (13)	0.0951 (15)	0.0183 (10)	0.0231 (10)	0.0537 (12)
C21	0.0554 (10)	0.0626 (11)	0.0613 (10)	0.0130 (8)	0.0128 (8)	0.0318 (9)
C22	0.1013 (19)	0.129 (2)	0.124 (2)	0.0219 (16)	0.0543 (16)	0.082 (2)
C23	0.0588 (9)	0.0370 (7)	0.0433 (8)	0.0151 (7)	0.0197 (7)	0.0176 (6)
C24	0.0669 (11)	0.0436 (9)	0.0646 (10)	0.0175 (8)	0.0323 (8)	0.0250 (8)
C25	0.1038 (16)	0.0482 (10)	0.0805 (13)	0.0213 (10)	0.0577 (13)	0.0311 (10)
C26	0.146 (2)	0.0545 (11)	0.0625 (12)	0.0292 (13)	0.0480 (14)	0.0356 (10)
C27	0.1183 (19)	0.0780 (14)	0.0636 (12)	0.0364 (13)	0.0173 (12)	0.0436 (11)
C28	0.0720 (12)	0.0676 (11)	0.0576 (10)	0.0244 (9)	0.0169 (9)	0.0355 (9)
C29	0.0635 (10)	0.0456 (9)	0.0530 (9)	0.0194 (8)	0.0279 (8)	0.0170 (7)
N1	0.0444 (7)	0.0371 (6)	0.0409 (6)	0.0132 (5)	0.0170 (5)	0.0168 (5)
N2	0.0506 (8)	0.0492 (7)	0.0468 (7)	0.0062 (6)	0.0148 (6)	0.0218 (6)
O1	0.0528 (6)	0.0585 (7)	0.0453 (6)	0.0001 (5)	0.0095 (5)	0.0236 (5)
O2	0.0826 (9)	0.0981 (11)	0.0548 (8)	0.0003 (8)	0.0104 (7)	0.0431 (8)

### Geometric parameters (Å, º)

**Table:**

C1—N1	1.4874 (17)	C14—H14C	0.9600
C1—C6	1.516 (2)	C15—O2	1.1872 (19)
C1—C2	1.551 (2)	C15—O1	1.3470 (19)
C1—H1	0.9800	C15—C16	1.486 (2)
C2—C3	1.504 (2)	C16—C21	1.384 (2)
C2—C12	1.5391 (19)	C16—C17	1.386 (2)
C2—H2	0.9800	C17—C18	1.383 (3)
C3—N2	1.274 (2)	C17—H17	0.9300
C3—C4	1.491 (2)	C18—C19	1.361 (3)
C4—C5	1.530 (2)	C18—H18	0.9300
C4—H4A	0.9700	C19—C20	1.387 (3)
C4—H4B	0.9700	C19—H19	0.9300
C5—N1	1.4696 (18)	C20—C21	1.389 (2)
C5—C23	1.5150 (19)	C20—C22	1.509 (3)
C5—H5	0.9800	C21—H21	0.9300
C6—C7	1.381 (2)	C22—H22A	0.9600
C6—C11	1.384 (2)	C22—H22B	0.9600
C7—C8	1.386 (2)	C22—H22C	0.9600
C7—H7	0.9300	C23—C24	1.380 (2)
C8—C9	1.365 (3)	C23—C28	1.381 (2)
C8—H8	0.9300	C24—C25	1.386 (3)
C9—C10	1.369 (3)	C24—H24	0.9300
C9—H9	0.9300	C25—C26	1.359 (3)
C10—C11	1.383 (2)	C25—H25	0.9300
C10—H10	0.9300	C26—C27	1.366 (3)
C11—H11	0.9300	C26—H26	0.9300
C12—C14	1.517 (3)	C27—C28	1.389 (3)
C12—C13	1.528 (3)	C27—H27	0.9300
C12—H12	0.9800	C28—H28	0.9300
C13—H13A	0.9600	C29—N1	1.4646 (19)
C13—H13B	0.9600	C29—H29A	0.9600
C13—H13C	0.9600	C29—H29B	0.9600
C14—H14A	0.9600	C29—H29C	0.9600
C14—H14B	0.9600	N2—O1	1.4524 (16)
			
N1—C1—C6	108.67 (11)	C12—C14—H14C	109.5
N1—C1—C2	111.65 (11)	H14A—C14—H14C	109.5
C6—C1—C2	111.47 (11)	H14B—C14—H14C	109.5
N1—C1—H1	108.3	O2—C15—O1	124.53 (15)
C6—C1—H1	108.3	O2—C15—C16	125.98 (15)
C2—C1—H1	108.3	O1—C15—C16	109.46 (13)
C3—C2—C12	117.24 (12)	C21—C16—C17	119.46 (16)
C3—C2—C1	106.81 (11)	C21—C16—C15	122.52 (15)
C12—C2—C1	114.42 (12)	C17—C16—C15	118.00 (15)
C3—C2—H2	105.8	C18—C17—C16	119.28 (19)
C12—C2—H2	105.8	C18—C17—H17	120.4
C1—C2—H2	105.8	C16—C17—H17	120.4
N2—C3—C4	127.95 (14)	C19—C18—C17	120.68 (19)
N2—C3—C2	119.25 (13)	C19—C18—H18	119.7
C4—C3—C2	112.59 (12)	C17—C18—H18	119.7
C3—C4—C5	108.92 (12)	C18—C19—C20	121.45 (18)
C3—C4—H4A	109.9	C18—C19—H19	119.3
C5—C4—H4A	109.9	C20—C19—H19	119.3
C3—C4—H4B	109.9	C19—C20—C21	117.63 (19)
C5—C4—H4B	109.9	C19—C20—C22	122.0 (2)
H4A—C4—H4B	108.3	C21—C20—C22	120.4 (2)
N1—C5—C23	112.04 (12)	C16—C21—C20	121.47 (18)
N1—C5—C4	110.59 (11)	C16—C21—H21	119.3
C23—C5—C4	108.34 (11)	C20—C21—H21	119.3
N1—C5—H5	108.6	C20—C22—H22A	109.5
C23—C5—H5	108.6	C20—C22—H22B	109.5
C4—C5—H5	108.6	H22A—C22—H22B	109.5
C7—C6—C11	117.98 (14)	C20—C22—H22C	109.5
C7—C6—C1	121.36 (14)	H22A—C22—H22C	109.5
C11—C6—C1	120.62 (13)	H22B—C22—H22C	109.5
C6—C7—C8	120.91 (17)	C24—C23—C28	118.47 (15)
C6—C7—H7	119.5	C24—C23—C5	120.19 (15)
C8—C7—H7	119.5	C28—C23—C5	121.24 (14)
C9—C8—C7	120.25 (17)	C23—C24—C25	120.61 (19)
C9—C8—H8	119.9	C23—C24—H24	119.7
C7—C8—H8	119.9	C25—C24—H24	119.7
C8—C9—C10	119.67 (17)	C26—C25—C24	120.70 (19)
C8—C9—H9	120.2	C26—C25—H25	119.6
C10—C9—H9	120.2	C24—C25—H25	119.6
C9—C10—C11	120.31 (18)	C25—C26—C27	119.22 (18)
C9—C10—H10	119.8	C25—C26—H26	120.4
C11—C10—H10	119.8	C27—C26—H26	120.4
C10—C11—C6	120.85 (16)	C26—C27—C28	120.9 (2)
C10—C11—H11	119.6	C26—C27—H27	119.5
C6—C11—H11	119.6	C28—C27—H27	119.5
C14—C12—C13	111.31 (16)	C23—C28—C27	120.04 (19)
C14—C12—C2	115.51 (14)	C23—C28—H28	120.0
C13—C12—C2	110.75 (14)	C27—C28—H28	120.0
C14—C12—H12	106.2	N1—C29—H29A	109.5
C13—C12—H12	106.2	N1—C29—H29B	109.5
C2—C12—H12	106.2	H29A—C29—H29B	109.5
C12—C13—H13A	109.5	N1—C29—H29C	109.5
C12—C13—H13B	109.5	H29A—C29—H29C	109.5
H13A—C13—H13B	109.5	H29B—C29—H29C	109.5
C12—C13—H13C	109.5	C29—N1—C5	108.29 (11)
H13A—C13—H13C	109.5	C29—N1—C1	108.89 (11)
H13B—C13—H13C	109.5	C5—N1—C1	114.03 (11)
C12—C14—H14A	109.5	C3—N2—O1	108.37 (11)
C12—C14—H14B	109.5	C15—O1—N2	113.56 (11)
H14A—C14—H14B	109.5		
			
N1—C1—C2—C3	−54.46 (15)	C16—C17—C18—C19	−1.4 (3)
C6—C1—C2—C3	−176.20 (12)	C17—C18—C19—C20	0.3 (3)
N1—C1—C2—C12	174.07 (12)	C18—C19—C20—C21	1.2 (3)
C6—C1—C2—C12	52.32 (16)	C18—C19—C20—C22	180.0 (2)
C12—C2—C3—N2	14.7 (2)	C17—C16—C21—C20	0.5 (3)
C1—C2—C3—N2	−115.22 (15)	C15—C16—C21—C20	178.90 (16)
C12—C2—C3—C4	−170.18 (13)	C19—C20—C21—C16	−1.6 (3)
C1—C2—C3—C4	59.93 (15)	C22—C20—C21—C16	179.6 (2)
N2—C3—C4—C5	113.23 (18)	N1—C5—C23—C24	−138.77 (14)
C2—C3—C4—C5	−61.40 (16)	C4—C5—C23—C24	98.96 (16)
C3—C4—C5—N1	55.67 (16)	N1—C5—C23—C28	44.96 (19)
C3—C4—C5—C23	178.82 (12)	C4—C5—C23—C28	−77.31 (18)
N1—C1—C6—C7	112.12 (15)	C28—C23—C24—C25	−0.7 (2)
C2—C1—C6—C7	−124.42 (15)	C5—C23—C24—C25	−177.07 (14)
N1—C1—C6—C11	−65.75 (16)	C23—C24—C25—C26	0.7 (3)
C2—C1—C6—C11	57.72 (17)	C24—C25—C26—C27	−0.3 (3)
C11—C6—C7—C8	0.7 (2)	C25—C26—C27—C28	0.0 (3)
C1—C6—C7—C8	−177.18 (15)	C24—C23—C28—C27	0.4 (3)
C6—C7—C8—C9	−1.1 (3)	C5—C23—C28—C27	176.73 (16)
C7—C8—C9—C10	0.2 (3)	C26—C27—C28—C23	0.0 (3)
C8—C9—C10—C11	1.0 (3)	C23—C5—N1—C29	63.82 (15)
C9—C10—C11—C6	−1.4 (3)	C4—C5—N1—C29	−175.21 (12)
C7—C6—C11—C10	0.5 (2)	C23—C5—N1—C1	−174.80 (11)
C1—C6—C11—C10	178.44 (15)	C4—C5—N1—C1	−53.82 (15)
C3—C2—C12—C14	−66.36 (19)	C6—C1—N1—C29	−61.46 (14)
C1—C2—C12—C14	59.87 (19)	C2—C1—N1—C29	175.18 (12)
C3—C2—C12—C13	61.33 (19)	C6—C1—N1—C5	177.49 (11)
C1—C2—C12—C13	−172.45 (14)	C2—C1—N1—C5	54.13 (15)
O2—C15—C16—C21	164.88 (19)	C4—C3—N2—O1	3.2 (2)
O1—C15—C16—C21	−16.9 (2)	C2—C3—N2—O1	177.53 (12)
O2—C15—C16—C17	−16.7 (3)	O2—C15—O1—N2	0.0 (2)
O1—C15—C16—C17	161.50 (15)	C16—C15—O1—N2	−178.20 (12)
C21—C16—C17—C18	1.0 (3)	C3—N2—O1—C15	169.28 (14)
C15—C16—C17—C18	−177.46 (16)		

### Hydrogen-bond geometry (Å, º)

Cg1 is the centroid of the C16–C21 ring.

**Table:**

*D*—H···*A*	*D*—H	H···*A*	*D*···*A*	*D*—H···*A*
C11—H11···*Cg*1^i^	0.93	2.95	3.778 (2)	149

Symmetry code: (i) −*x*+2, −*y*+1, −*z*+1.
